# Description of a Modified Two-Step Omphalectomy Technique Using the LigaSure^™^ Device to Remove the Whole Extrahepatic Umbilical Vein: A Case Series Study in Equine and Donkey Foals

**DOI:** 10.3390/ani15070981

**Published:** 2025-03-28

**Authors:** Antonio Buzon-Cuevas, Juan Duaso, Antonia Sanchez de Medina, Juan M. Sierra, Alejandro Perez-Ecija, Francisco J. Mendoza

**Affiliations:** 1 Department of Animal Medicine and Surgery, University of Cordoba, 14014 Cordoba, Spain; vetbuzon@gmail.com (A.B.-C.); juanduaso@gmail.com (J.D.); tsmedina189@hotmail.com (A.S.d.M.); juanmasierra@hotmail.es (J.M.S.); fjmendoza@uco.es (F.J.M.); 2 Veterinary Teaching Hospital, University of Cordoba, 14014 Cordoba, Spain

**Keywords:** donkey, omphalitis, septic foal, umbilical vein abscess, urachus

## Abstract

Umbilical disorders are common in equids, although scarce information is available in donkeys compared to horses. Foals with these disturbances have high morbidity and mortality rates. The conventional omphalectomy technique does not remove the whole umbilical vein, which can lead to infection in the remnant vein or even liver abscess. At the moment, if the whole umbilical vein needs to be removed, a longer ventral midline incision must be performed, which is linked with several complications. This retrospective case series study focused on a modified two-step omphalectomy using the LigaSure^TM^ device in each step in five neonate foals (4 horses and 1 donkey) with umbilical disorders. Using this modified technique, the whole umbilical vein (from the stump to the liver) can be removed without the need of incision extension. Two foals in this series had complications not related with the surgery and had to be euthanized. In the surviving foals (three of five), no long-term complications have been reported. Therefore, this modified two-step technique using the LigaSure^TM^ device could be a suitable surgical option to decrease short- and long-term complications in neonate foals undergoing umbilical surgery. However, a multicentric prospective study on a larger number of animals is required.

## Simple Summary

Umbilical disorders are common in equids, showing high morbidity and mortality. Short- and long-term complications with the conventional omphalectomy technique for stump removal have been described. This study focused on a modified two-step omphalectomy technique using the LigaSure^TM^ device, which allows removal of the whole umbilical vein. Four horse foals and one donkey foal (less than one week old) with different umbilical disorders were included. Short- or long-term complications related to this novel modified surgical technique were not observed. However, two horse foals were euthanized because of septic arthritis or peritonitis secondary to a foreign body-related small-intestine rupture. This modified technique could decrease post-operative complications such as umbilical vein or hepatic abscesses, reducing the risk of future complications, reinterventions, and hospitalizations.

## 1. Introduction

In neonatal equids, umbilical disorders are a common problem (prevalence of 5%) [1]. Although no epidemiological information is available on donkey neonates, these conditions are assumed to be more frequent in this species [2]. Foals with umbilical disorders are at higher risk of hematogenous dissemination of infection and developing sepsis or other complications such as diarrhea, pneumonia, septic physitis, or arthritis, with deleterious effects on future performance and survival of the affected animals [3].

Omphalophlebitis is the most common disturbance of the internal umbilical remnant, although other disorders such as patent urachus, urachal rupture with secondary uroperitoneum, omphaloarteritis, umbilical hernia, and omphalitis (without vessels involvement) are also observed [3]. Periumbilical hematoma, urachal diverticula, or urachal cysts are other less frequently reported disturbances [3].

Omphalophlebitis is a bacterial infection of the umbilical vein, commonly acquired via ascendant colonization through the umbilical stump in the first week of life [4]. Poor hygienic environmental conditions, congenital anatomical abnormalities, and any primary disease causing a failure of the passive transfer of immunity (such as maladjustment neonatal syndrome, sepsis, prematurity, and immaturity) are considered risk factors for omphalophlebitis, both in horse and donkey foals [2,5,6]. Moreover, some practices such as stump manipulations, ligation, chemical cauterization, and direct application of repellents or disinfectants can also irritate the stump and contribute to omphalophlebitis [4]. Foals with omphalophlebitis can also develop hepatic abscesses, increasing the mortality rate [7].

Medical treatment based on broad-spectrum systemic antibiotics is usually the first choice [2,6,8]. Surgery is usually an elective (non-emergency) procedure and/or conducted when the response to the initial medical treatment alone is unsatisfactory [1]. Omphalectomy via a ventral midline laparotomy is commonly performed in foals with omphalophlebitis [9]. In order to remove the whole vein, a larger ventral incision is necessary, which is linked with high odds of complications [10,11]. Midline or paramedian marsupialization of the umbilical vein (either alone or combined with a conventional omphalectomy) could be performed in these animals [12]. Laparoscopic resection of the internal umbilical structures is rarely performed in horse foals [7,13], and no information on donkeys is available. A modified two-step omphalectomy technique where both the main internal umbilical structures (first step) and the remaining cranial umbilical vein (second step) are removed without extending the ventral incision could be a solution.

LigaSure™ (LigaSure Atlas™, Covidien Medtronic, Minneapolis, MN, USA) is a bipolar electrocautery device used for vessel sealing and tissue dissection, able to reduce bleeding, thermal tissue damage, surgery time, and risk of infection [14]. Despite this device being idoneous to remove the whole umbilical vein using a small ventral incision, there are no reports describing this modification of conventional omphalectomy in horse foals. In addition, to the authors’ knowledge, there are no studies reporting the use of the LigaSure™ device in donkey foals.

Our hypothesis is that a modified two-step omphalectomy technique using the LigaSure™ at each step could be suitable in these foals, avoiding the need for marsupialization of the umbilical vein or a large ventral incision. This technique would reduce short- and long-term complications, time of hospitalization, and veterinary expenses.

## 2. Materials and Methods

### 2.1. Animals

The following inclusion criteria were used: foals referred to the Veterinary Teaching Hospital (VTH) of the University of Cordoba along 2024 with umbilical disorders such as omphalitis, omphalophlebitis, omphaloarteritis, persistent urachus, and umbilical vein or hepatic abscess that underwent a modified two-step omphalectomy technique using the LigaSure™ device. This modified two-step technique was carried out in all the foals included in this case series. The exclusion criteria were the following: foals with umbilical infection that were treated medically or that were euthanized before surgery (economic restraints, poor prognosis due to concomitant disorders, etc.), foals that underwent umbilical resection without evidence of infection, and those that were operated on by other surgical teams using different techniques.

Data collected included signalment (species, breed, age, gender), history (prematurity, etc.), administration of medications prior to admission, physical examination, laboratory findings, ultrasonographic findings, surgical findings (duration of surgery, affected structures, etc.), additional procedures (arthroscopy or joint lavage, culture results, etc.), pre- and post-operative management, short- and long-term complications, and survival.

### 2.2. Umbilical Disorder Diagnosis

Diagnosis of umbilical infection was based on a combination of physical examination, ultrasonographic evaluation, and surgical findings. Abdominal ultrasounds were performed in every foal on admission to assess the presence of abnormalities in the umbilical structures (umbilical vein and arteries, urachus, and internal umbilical remnant) with a 3.5 MHz microconvex or 10 MHz linear transducers (MyLab 50 XVision, Esaote, Barcelona, Spain).

The disorders were classified according to the affected structures using previously reported species-specific ultrasonographic measurements [15,16,17]: umbilical vein abscessation, hepatic abscessation (when the abscess extended from the umbilical vein to the liver parenchyma), omphalophlebitis (umbilical vein wider than 10 mm in horses or 8 mm in donkeys), omphalitis (internal umbilical remnant, with/without urachitis, wider than 25 or 22 mm in horse or donkey foals, respectively), omphaloarteritis (umbilical artery wider than 10 mm in horses or 8 mm in donkeys), patent urachus, and external umbilical infection or abscessation.

### 2.3. Surgical Procedure

All foals were anesthetized following a similar partial intravenous anesthesia (PIVA) protocol with minor variations depending on the primary disorder [18]. Briefly, dexmedetomidine, midazolam, methadone, and fentanyl were used as premedication, and induction was carried out with propofol alone or combined with ketamine. Maintenance was carried out with inhalant isoflurane and glucose 5% in Ringer’s lactate solution (5 mL/kg/h) in all foals, and some of them required dobutamine as vasopressor therapy or dexmedetomidine to improve the anesthetic plane and recovery. Doses, products, and manufacturers are listed in Table S1.

The modified technique consisted of two steps: the first a conventional omphalectomy with cystoplasty, and the second the removal of the whole umbilical vein up to the liver. The LigaSure™ device was used in both steps.

**Figure 1:**
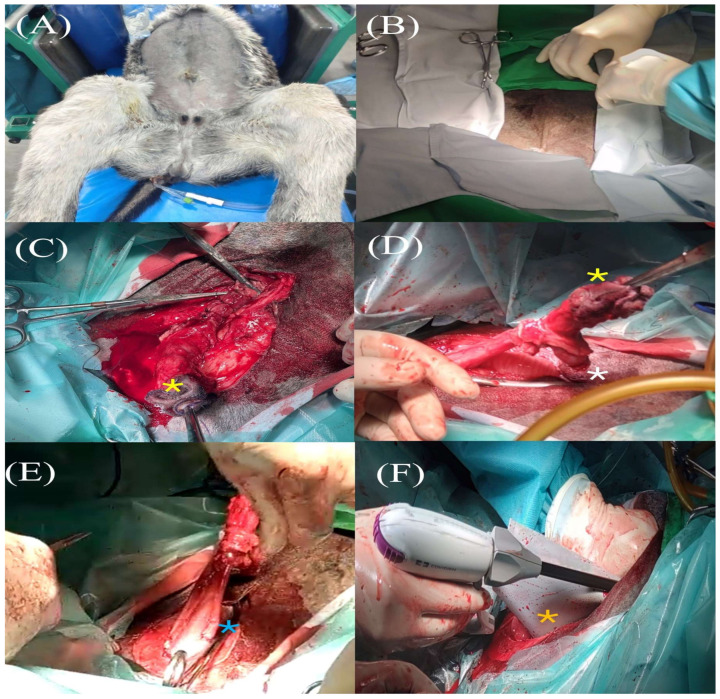
Modified two-step omphalectomy technique description. (**A**) Donkey foal placed on dorsal recumbency. (**B**) Aseptically prepared surgical area. (**C**) Double ligation of the umbilical vein cranial to the stump prior to vein transection using LigaSure™. Note that the stump has been grasped with forceps (yellow asterisk). (**D**) Dissection of the abdominal wall in order to isolate the internal umbilical structures and bladder while the stump is grasped (yellow asterisk). Note the previously transected umbilical vein has been clamped to the abdominal wall to keep the tension (white asterisk). (**E**) Double ligation of the left umbilical artery prior to artery transection with the Ligasure™ device (blue asterisk). (**F**) Both LigaSure™ and the surgeon’s hand are placed inside the abdominal cavity to remove the entire umbilical vein. Note the silicone sheet protecting the abdominal viscera (orange asterisk).

Conventional omphalectomy was performed as previously described [10,11]. Briefly, foals were placed in dorsal recumbency, and a urinary catheter was placed. The umbilical stump was isolated to avoid any contamination, with a 1 USP non-absorbable synthetic monofilament suture (Dafilon^®^, BBraun Vet Care, Barcelona, Spain) in a continuous inversion pattern (Figure 1A). Once the surgical area was aseptically prepared (Figure 1B), a fusiform skin incision around the stump was performed with a monopolar electric scalpel (es300, EMED, Opacz-Kolonia, Pruszków, Poland) (Figure 1C). The umbilical vein was carefully externalized using a 2 cm incision cranial to the stump, and double ligated (2-0 USP absorbable synthetic monofilament sutures (Monosyn^®^, BBraun Vet Care, Barcelona, Spain)) to prevent abdominal cavity contamination (Figure 1D). Then, the LigaSure™ device was used to transect the vein between both sutures. The umbilical vein remnant was clamped to the abdominal wall to keep it in tension (Figure 1E).

Afterwards, the stump was grasped and traction applied to expose the rest of umbilical structures (Figure 1E). The abdominal wall around it was sharply dissected using curved Metzenbaum scissors, avoiding damage to the internal umbilical structures and abdominal viscera. Both umbilical arteries were double ligated and transected in a similar way to the umbilical vein. This first step included a conventional cystoplasty, which was performed as previously described [19]. Briefly, the vesical trigone was clamped with atraumatic Doyen intestinal forceps, transected, and sutured (2-0 USP Monosyn^®^, BBraun Vet Care, Barcelona, Spain) using the Parker–Kerr pattern.

The second step of this modified technique is the removal of the remaining umbilical vein. First, the midline incision was extended cranially approximately 3 cm (enough for the surgeon’s hand to be introduced inside the abdominal cavity) and a blunt dissection of the remaining umbilical vein was performed up to the liver. A silicone sheet was placed between the abdominal viscera and the abdominal wall. Then, the surgeon’s hand was introduced with the palm facing down (forming a concave shape adapted to the liver surface) and the umbilical vein was held between the fingers. The LigaSure™ device was then used to transect the vein at the most cranial point (closest to the hand, which protected the liver) (Figure 1F, Video S1). The whole vein was then extracted.

After completing both steps, the linea alba was closed with a 2 USP coated synthetic absorbable monofilament polyglycolic acid suture (Safil^®^, BBraun Vet Care, Barcelona, Spain) in a simple continuous pattern, the subcutaneous tissue using a simple continuous pattern (2-0 USP Monosyn^®^, BBraun Vet Care, Barcelona, Spain), and the skin in a simple interrupted pattern (0 USP Monosyn^®^, BBraun Vet Care, Barcelona, Spain). A stent was placed to protect the incision and sutured to the skin with a 0 USP synthetic monofilament non-absorbable suture (Dafilon^®^, BBraun Vet Care, Barcelona, Spain), and the abdomen was bandaged using elastic adhesive tape (Tensoplast^®^, BSN medical Inc., Bedfordshire, UK). The stent was removed 24–72 h post-surgery depending on dirtiness and correct fitting. Sterile gauzes were placed over the incision in further bandages. The sutures were removed 14 days after surgery.

Post-operative medications (antibiotics, anti-inflammatory, pain relief, etc.) were administered to all foals according to the internist’s preference and concurrent diseases. Further information is provided in the case description.

### 2.4. Outcome and Follow-Up

Short-term post-operative complications were defined as those complications either related (incisional infection, herniation, etc.) or non-related (septic arthritis, diarrhea, pneumonia, etc.) with the surgical technique that occurred at the time of discharge from the hospital. Long-term post-operative complications were defined as any disturbance which occurred after discharge. The follow-up was performed by phone calls to the owners and referring veterinarians.

All foals were evaluated daily during hospitalization, both before and after the surgery, since they were housed in the neonatology intensive care unit. This monitoring consisted in (at least) checking of the vital signs and physical examination (suckling reflex, joints, etc.), routine blood work (hematology and plasma glucose, lactate, creatinine, IgG, and electrolyte concentrations), and daily thoracic and abdominal ultrasounds, in order to determine any short-term complication and worsening.

### 2.5. Statistical Analysis

The study design was a retrospective descriptive case series. The descriptive analysis was performed using specialized commercial statistical software (IBM SPSS Statistics 28, IBM, Chicago, Illinois). Doses, products, and manufacturers of drugs used for treatments are listed in Table S1.

## 3. Results

A total of five neonates were included in the study—four horse foals (two male and two female) and one female donkey foal—1–6 days old and weighing 52–66 kg. Three of the foals were Andalusian and one was Hanoverian, whereas the donkey foal was Andalusian.

The following umbilical disturbances were included: omphalophlebitis without abscess formation (n = 1, case 4), omphalophlebitis with umbilical vein abscess (n = 1, case 1), hepatic abscess after conventional omphalectomy (n = 1, case 5B), omphaloarteritis (n = 2, cases 3 and 5A), and omphalitis without umbilical vessel involvement (n = 1, case 2). Three foals (60%) also had a concurrent patent urachus (Table 1 and Table 2).

No short-term complications related to the modified two-step omphalectomy technique were detected in any foal, although complications related to concurrent systemic diseases such as enteritis, diarrhea, peritonitis, and arthritis were noted in four animals (80%) (Table 2).

The survival rate was 60% (three of five). One foal died due to a small-intestine rupture secondary to a foreign body. Another foal developed a septic polyarthritis caused by a multiresistant bacterium, and the owners decided to euthanize it due to the poor prognosis for future athletic performance. No long-term complications related to the modified technique were noted in the surviving animals (Table 2).

### 3.1. Case Descriptions and Perioperative Managements

#### 3.1.1. Clinical Case 1

A six-day-old Andalusian female donkey foal (weighing 52 kg) was referred to the VTH due to persistent patent urachus and inflammation of the stump. The donkey foal had been treated by her veterinarian with intravenous flunixin meglumine and intramuscular cefquinome twice daily, several intravenous bolus of saline solution with glucose, a 1 L plasma transfusion from her mother (serum IgG concentration < 400 mg/dL), and local rinse of the stump with chlorhexidine several times daily. Blood analysis (prior to referral) showed mild leukocytosis (13.7 × 10^3^/µL), neutrophilia (10.7 × 10^3^/µL), lymphopenia (0.9 × 10^3^/µL), monocytosis (1.9 × 10^3^/µL), thrombocytosis (381 × 10^3^/µL), and hypertriglyceridemia (187 mg/dL). The rest of the biochemical parameters (creatinine, lactate, and liver enzyme concentrations) were within normal ranges. Delivery was uneventful and on the estimated date, but the jenny rejected the foal (Table 1).

On admission, the neonate was bright and alert, with normal sucking reflex, and no joint disturbances were noticed. The stump was wet and thicker than normal, with moderate pain on palpation. The rest of the physical examination was normal. Hematology showed lymphopenia and monocytosis (Table 3). Biochemistry parameters (IgG, creatinine, lactate, and liver enzyme concentrations) were within normal ranges (Table 3).

**Figure 2:**
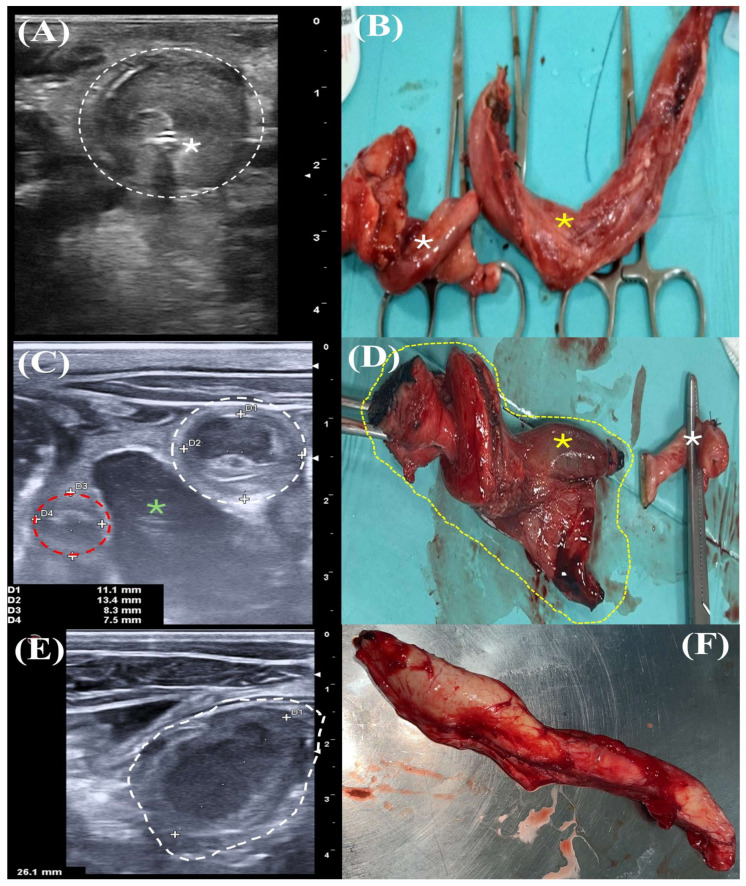
Ultrasound and macroscopic views of the internal umbilical structures removed using the modified two-step omphalectomy. (**A**) Internal umbilical remnant ultrasound showing an enlarged umbilical vein (higher than 1 cm, dotted white line) with a hyperechogenic foci displaying acoustic shadow (white asterisk). (**B**) Enlarged abscessed umbilical vein (yellow asterisk) in a donkey foal. Compare the size of the umbilical vein (yellow asterisk) with the rest of the internal umbilical stump and the bladder apex (white asterisk). (**C**) Internal umbilical remnant ultrasound showing an enlarged umbilical artery (greater than 1 cm, white dotted line) compared to a normal-sized and ultrasonographic artery (red dotted line). Bladder and urachal communication (patent urachus) can also be seen in the image (green asterisk). (**D**) Macroscopic view of an enlarged, congestive, and edematous umbilical artery (yellow asterisk). Note the external stark and bladder apex (yellow spotted line) and an umbilical vein of normal size and appearance (white asterisk). (**E**) Ultrasound of the abdominal cavity showing a rounded mass (approx. 2.6 cm) close to the liver displaying an echogenic wall with an hypoechogenic content compatible with a liver abscess (white dotted line). (**F**) Macroscopic image of the abscessed umbilical vein remnant forming an abscess in the entry of the liver.

The abdominal ultrasound, based on previously reported species-specific data in donkey foals [17], revealed normal-sized umbilical arteries, a completely open urachus with communication between the urinary bladder and the stump, enlargement (approx. 2.5 cm) and edema of the internal umbilical remnant, and an increase in the size of the umbilical vein (approx. 1.5 cm) affecting the entire vein from the stump to the liver, with a hyperechogenic content compatible with an umbilical vein abscess (Figure 2A). No other abnormality was observed in the abdomen. Several pneumonic foci (ranged between 0.5–1.5 cm) were observed on both hemithoraces. The foal was diagnosed with omphalophlebitis with umbilical vein abscess and persistent patent urachus, with a good-to-guarded prognosis.

A modified two-step omphalectomy technique using the LigaSure™ at each step was performed the same day (Figure 1 and Figure 2B). Anesthesia was uneventful and recovery was normal. Surgery duration was 75 min. Systemic ampicillin, amikacin, and flunixin meglumine were administered for three days after the surgery. The foal was allowed to nurse freely, and glycemia was checked every 2 h. The donkey was discharged after 3 days of hospitalization and prescribed with antibiotic for 5 additional days, abdominal bandage, and full rest in the box for 12 additional days (Table 2). Sutures were removed 15 days after the surgery by her veterinarian. Phone follow-ups with owner and veterinarian have yielded no short- or long-term complications (10 months old).

#### 3.1.2. Clinical Case 2

A 12-h-old Andalusian female foal (weighing 54 kg) was referred due to weakness. Previous laboratory analysis showed leukopenia (3.7 ×10^3^/µL) and high serum creatinine (5.3 mg/dL) and lactate (8.8 mmol/L) concentrations. Delivery was normal and on date. Meconium was passed, but urination was not observed. Flunixin, meglumine, and cefquinome were administered prior to submission.

On admission the foal was weak with low maternal interest, decreased response to external stimuli, and weak sucking reflex. During physical examination, increased respiratory (92 rpm) rate, fever (39.5 °C), and congestive mucous membranes were noted (Table 1). The foal was assisted to nurse colostrum. The blood profile showed moderate leukopenia, neutropenia, lymphopenia, and monocytosis, increased serum lactate and creatinine concentrations, and low serum IgG concentration (Table 3).

The abdominal ultrasound showed a heterogenous internal umbilical remnant of normal size [16]. No other abnormality was noticed. The foal was diagnosed with sepsis, total failure of transfer of passive immunity (FTPI), and omphalitis, with a guarded-to-poor prognosis (Table 2).

Intravenous ampicillin, meloxicam, a 2 L plasma transfusion (from a compatible horse donor from the VTH plasma bank), and fluid therapy using Isofundin^®^ at 6 mL/kg/h supplemented with 40% glucose (BBraun Vet Care, Barcelona, Spain) were administered. The foal was allowed to nurse freely and was housed in the neonatal critical care unit for close monitoring.

Twelve hours after admission, the left hindlimb hock appeared distended. Arthrocentesis showed high leukocyte counts (48.6 × 10^3^/μL), 72.1% lymphocytes, 22.7% neutrophils, and a total solid concentration of 5 g/dL, without degenerative changes in neutrophils or pathogens on cytological evaluation. One day later (36 h after admission), additional joints (stifle and fetlock of the left hindlimb, as well as the contralateral hock) were also affected. Leukocyte counts and total solid concentrations were 4–24 × 10^3^/μL and 4.5–6.1 g/dL, respectively. Since an arthroscopy-guided joint lavage of every affected joint was decided upon, a modified two-step omphalectomy technique using LigaSure^TM^ was also performed under the same general anesthesia. Anesthesia and recovery were uneventful. Surgery duration was 80 min. Amikacin was administered in each joint prior to port closure. A culture of the synovial fluid from the affected joints did not yield bacterial growth, and thus immune-mediated polyarthritis was confirmed.

Intravenous amikacin was added to the medical treatment, and meloxicam was changed to flunixin meglumine. The abdominal bandage was removed two days after surgery. The incision had a good external appearance, and no ultrasonographic abnormalities were found. The foal was discharged 4 days after admission due to financial constraints with abdominal and joint bandages and full rest in the box for 15 additional days. No short-term complications related to the omphalectomy were reported during hospitalization, although the foal had a couple of days of soft feces lasting 48 h after the surgery. After discharge, an incisional seroma was observed for some days, which resolved with bandages. No surgery-related long-term complications have been observed at this time, and the foal is still alive (Table 2).

#### 3.1.3. Clinical Case 3

A 12 h-old Andalusian male foal (weighing 53 kg) was referred to the VTH due to severe weakness and incapacity to stand by himself. The foal did not ingest any colostrum due to mare agalactia, and urine and meconium were not passed. The delivery was two weeks earlier than the estimated date and the labor was assisted, lasting 15–25 min.

On admission, the foal was weak, with poor sucking reflex and response to external stimuli, congestive tongue and mucous membrane, and prolonged capillary refill time (approx. 2–3 s). The stump was hemorrhagic, and flexural deformity in the right hindlimb was noted (Table 1).

The blood profile showed neutrophilia and lymphopenia, increased serum lactate concentration, creatinine in the upper limits, and low serum IgG concentrations (Table 3). Umbilical ultrasounds revealed an increase in the size of the internal umbilical remnant (approx. 2.7) and both umbilical arteries (approx. 1–1.5 cm), with a hyperechogenic pattern and blood flow in the interior of the right artery [15]. Hypoechogenic rounded images were observed in the small colon (Table 2). Radiographs of both carpi showed normal bone ossification. The foal was diagnosed with neonatal maladjustment syndrome, prematurity, total FTPI, omphaloarteritis, and meconium retention, with a guarded-to-poor prognosis.

Systemic medical treatment was established based on ampicillin, amikacin, flunixin meglumine, thiamine, magnesium sulfate, vitamin E, selenium, plasma transfusion (2 liters from a compatible horse donor from the VTH plasma bank), and fluid therapy (Ringer lactate^®^ supplemented with glucose 40% (BBraun Vet Care, Barcelona, Spain)) at 6 mL/kg/h. A nasogastric tube was placed, and a milk replacement was administered hourly through the probe. A rectal enema was administered every 4 h. A constant-rate infusion of furosemide diluted in a 5% glucose solution (BBraun Vet Care, Barcelona, Spain) was administered for 24 h in order to increase urine production. The foal was housed in the neonatal critical care unit for close monitoring. The response to treatment was good, with the animal standing by himself and passing meconium at 24 h from admission.

Despite this positive evolution during the first days, daily abdominal ultrasounds showed heterogeneous internal umbilical remnant and enlarged umbilical arteries (approx. 1–1.5 cm) (Figure 2C); thus, a modified two-step omphalectomy using the LigaSure^TM^ was performed 6 days after admission (Figure 2D, Video S1). Anesthesia and recovery were uneventful. Surgery duration was 90 min. Post-surgical medical treatment was based on ampicillin, amikacin, and flunixin meglumine. A constant-rate infusion of lidocaine was initiated 24 h after surgery due to hypomotility, mild distention of the small intestine, and soft feces. Five days after surgery, the foal showed acute high fever (39.5 °C), severe weakness, and ileus (Table 2). Blood analysis revealed severe leukopenia (2.7 × 10^3^/μL) and neutropenia (1.9 × 10^3^/μL). Abdominal ultrasound showed great amounts of hyperechogenic free fluid. Abdominocentesis was performed, and a diagnosis of septic peritonitis (WBCs 34.6 × 10^3^/μL, total protein 3.2 g/dL, lactate 6.4 mmol/L, creatinine 1 mg/dL) was made. An emergency exploratory laparotomy was performed, where a perforation of the proximal jejunum due to a stick of shaving and large quantities of shavings (most likely from the bedding) inside the small and large intestines were found. Euthanasia was performed due to the poor prognosis.

#### 3.1.4. Clinical Case 4

A 12 h-old Andalusian female foal (weighing 53 kg) was referred due to severe weakness and incapacity to stand by herself. No colostrum had been ingested, and neither urine nor meconium had been passed. The delivery was 21 days earlier than expected, and the labor was not assisted. Three neonate foals (less than 48 h of life) had died and several abortions had been noted on the farm during the season due to respiratory problems. The mare had no history of equine herpesvirus 1 or 4 vaccination during the pregnancy (Table 1).

On admission, the foal was ambulant, bright, alert, and responsive, with a good sucking reflex. Severe fever (39.7 °C), tachypnea (96 rpm), tacky mucous membranes, and a prolonged capillary refill time (2–3 s) were observed. No abnormalities of the stump or joints were detected. The blood analysis showed left shift and lymphopenia, azotemia, and high serum lactate concentrations (Table 3). Arterial blood gas analysis revealed metabolic acidosis (pH 7.25; bicarbonate 15.1 mmol/L, P_a_CO_2_ 39 mmHg) and hypoxemia (P_a_O_2_ 80 mmHg, SO_2_ 93%). Retained meconium and pleural hyperechogenic irregularities (B lines on both lungs) were also observed during the abdominal and thoracic ultrasounds, respectively. Thoracic radiography showed a severe interstitial pulmonary pattern. Radiographs of both carpi showed normal bone ossification. The foal was diagnosed with acute respiratory distress due to severe interstitial pneumonia (likely viral), sepsis, prematurity, omphalophlebitis, and meconium retention, with a poor prognosis.

Systemic medical treatment was established based on ampicillin, dexamethasone (only one dose), meloxicam, continuous oxygen therapy, inhaled fluticasone, inhaled salbutamol, partial parenteral nutrition, and fluid therapy with Ringer lactate^®^ supplemented with 40% glucose (BBraun Vet Care, Barcelona, Spain) at 6 mL/kg/h. A constant-rate infusion of furosemide diluted in a 5% glucose solution (BBraun Vet Care, Barcelona, Spain) was administered for 36 h. Maternal milk was administered hourly through a nasogastric tube. A rectal enema was administered every 4 h. The foal was housed in isolation in the neonatal critical care unit for close monitoring.

During the next five days, the evolution was satisfactory: respiratory rate decreased, acid–base disturbances improved, oxygen-blood content increased, and both serum creatinine and lactate concentrations decreased. Therefore, fluticasone and salbutamol were withdrawn. Since the foal began to tolerate higher volumes of milk, the nasogastric tube was removed. Serial abdominal ultrasounds showed a worsening of the umbilical structures, with increased umbilical vein size from the liver to the stump (ranging 1–1.5 cm), a full open urachus, and mild small-intestine distention. Oral lactase and misoprostol were added to the initial medical treatment. A modified two-step omphalectomy was performed 7 days after admission. The surgery duration was 80 min. Treatment after surgery was based on ampicillin, amikacin, and flunixin meglumine. The foal was discharged 5 days after surgery without any medical treatment, with only abdominal bandage and full rest in the box. No short-term complications were recorded during post-surgery hospitalization or at home. The suture was removed five days after discharge (Table 2). Phone follow-ups with the owner and the veterinarian have indicated no short- or long-term complications (7 months-old).

#### 3.1.5. Clinical Case 5

A 16-h-old Hanoverian male foal (66 kg) was referred due to colic signs and a hemorrhagic stump. The delivery was on date and without complications. The foal had an ingested colostrum, and urine but no feces had been passed. On admission, the foal was alert, bright, and responsive, with a normal sucking reflex and vital signs. However, mild colic signs (flank watching, abnormal behavior, alternate recumbency and standing), several laborious unproductive attempts to defecate, and the presence of active hemorrhage from the umbilical stump (mostly during attempts to defecate) were noticed (Table 1).

Blood work analysis showed leukopenia, neutropenia, lymphopenia, increased serum lactate concentrations, and serum IgG concentrations above the cut-off limits (Table 3). Abdominal ultrasound showed several hypoechogenic rounded masses in the small colon, gas distention of the large bowel, hypomotility of the small intestine with mild distention, communication between the bladder and the stump, a slightly larger-than-normal left umbilical artery (1.2 cm), increased (>2.5 cm) and edematous internal umbilical remnant [15], and a large amount of hypoechogenic peritoneal free fluid. Abdominal radiography showed an image compatible with meconium retention and abundant free peritoneal fluid. Abdominocentesis yielded a cloudy serohemorrhagic peritoneal fluid with high leukocyte counts (49.1 × 10^3^/µL), total proteins (4.8 g/dL), and lactate (7.9 mmol/L) concentrations, with a peritoneal creatinine concentration within normal limits (1.7 mg/dL). No gastric reflux was retrieved. The foal was diagnosed with acute colic due to meconium impaction, omphaloarteritis, and persistent patent urachus, with a guarded prognosis. Thus, a laparotomy under general anesthesia for colic surgery and a conventional omphalectomy were performed.

Anesthesia, surgery, and recovery were normal. Peri-operative treatment was based on ampicillin, amikacin, flunixin meglumine, thiamine, magnesium sulfate, vitamin E, selenium, misoprostol, an intravenous bolus of 1 L Ringer lactate^®^ every 3 h supplemented with 40% glucose (BBraun Vet Care, Barcelona, Spain), free nursing, and an abdominal bandage. Twenty-hours post-surgery, the abdominal bandage was removed due to hyperexcitation and anxious and aggressive behavior against the bandage. The incision was then covered with an adhesive tape (Snögg Adhesive Foam Bandage, Snögg Animal Care, Barcelona, Spain). The foal was discharged 5 days after admission, with a recommendation of full rest in a small box with the mother. However, 2 days after discharge, the foal escaped from the box and ran out in the paddock, causing an incisional herniation. The referral veterinarian reintroduced the omentum and sutured the incisional stoma prior to referring the foal again.

On second admission, the foal was directly sent to the operating room, where the omentum was partially removed, the abdominal cavity was gently rinsed with gentamicin and large volumes of Ringer lactate^®^ (BBraun Vet Care, Barcelona, Spain), and the abdominal wall was sutured. Surgery duration was 90 min. Treatment was based on ampicillin, amikacin, flunixin meglumine, a constant rate infusion of lidocaine, and a bandage of the abdominal cavity. Four days after the second surgery, multiple diffuse hyperechogenic points were observed along the incision, and dehiscence of one of the simple sutures was noticed. Antimicrobials were changed to sulfadoxine–trimethoprim, metronidazole, and florfenicol. During the following five days, several joints (right forelimb fetlock and carpal, and left hock and stifle) appeared warm and distended, and the abdominal ultrasound showed worsening of the incision with hyperechogenic subcutaneous fluid (2–3 cm) and an enlarged (1.8 cm) umbilical vein remnant entering the liver with hyperechogenic content compatible with a liver abscess (Figure 2E). The foal was sent to surgery in order to remove the remaining vein using the LigaSure™ device (Figure 2F), incision debridement and cleaning, and lavage of affected joints by arthroscopy. No complications related to the technique were noticed in the short term. However, despite additional joint lavages by needle and arthroscopy with different antibiotics, the septic polyarthritis became worse, and cultures of synovial fluid and exudate showed growth of a multiresistant Gram-positive bacterium (Table 2). Due to the poor prognosis, facility biohazard concerns, and current European antibiotic stewardship, the foal was euthanized (34 days old).

## 4. Discussion

Umbilical disorders are common in neonatal foals, and can lead to systemic diseases such as sepsis, septic polyarthritis, diarrhea, and pneumonia, impairing the prognosis and outcomes such as future athletic performance. Since the umbilicus constitutes the main entry point for pathogens at this stage of life, it should be closely evaluated and monitored. This case series study describes a modification of the conventional omphalectomy technique in a modified two-step technique aimed at easier access and removal of the whole umbilical vein. This new two-step technique avoids the risk of future complications such as abscess of the umbilical vein remnant, liver abscesses, and due to the use of the LigaSure™ device, requires a smaller ventral midline incision compared to other techniques, decreasing the odds of short- and long-term incisional complications.

This study is also the first to describe this modified omphalectomy in a donkey neonate (as can be seen in Figure 1). Moreover, and despite the fact that donkeys, like horses, also suffer from umbilical disorders [2], this study is also pioneering in describing the clinical presentation, ultrasound findings, and macroscopic pathology of an umbilical vein abscess in this species (Figure 2A,B).

The conventional omphalectomy technique involves the ligation of the umbilical arteries, the urachus, and the umbilical vein (and a cystoplasty in animals with concurrent patent urachus) [9,19]. In this technique, although the surgical opening is enough to extract most of the internal umbilical structures, the most cranial part of the umbilical vein (up to the liver) cannot be removed. Thus, a vein remnant remains inside the abdomen that if previously infected can later become an abscess, with the subsequent risk of complications and requiring a second surgery to remove the infection foci. This could be associated with longer hospital stays, higher odds of short-term complications, and increased veterinary costs.

In those cases where an enlarged umbilical vein (with or without liver abscess) is noticed, the conventional omphalectomy requires a cranial extension of the original incision to broaden the surgical field to improve the approach for vein ligation and resection [12]. This extended incision increases the probability of short- or long-term complications such as infection, eventration, hernias, or adhesions [10,11,23]. In order to avoid making a long midline incision, conventional omphalectomy can be combined with marsupialization; however, it has been recently reported that this procedure also increases the probability of short-term complications such as herniation and stoma infection [24,25].

Although laparoscopy could avoid these complications, it is rarely performed in equine foals [7,13], and the technique for a complete en bloc resection of all umbilical structures, even including the apex of the bladder, has not been described in horses or donkeys [26]. Moreover, although a laparoscopically assisted technique would increase safety and precision, our assisted sealing technique is less technically complex, requiring less expensive equipment and reducing surgery and anesthesia duration.

The authors propose a modification of the conventional omphalectomy, which allows the resection of the whole umbilical vein using the LigaSure^TM^ device. With this modified technique, no umbilical remnants are left in the abdomen, decreasing the risk of future complications, which could compromise the animal’s life or impair the outcome.

Since this modified two-step technique uses the LigaSure^TM^ device, the cranial extension of the incision is smaller (approx. 5 cm, enough for introducing the hand and the device) compared to the one needed for complete resection of the umbilical vein or liver abscesses using the conventional technique (approx. 5–15 cm), given a final incision length shorter with our technique.

The LigaSure™ device is gaining popularity in equid veterinary medicine, with many surgeons using it to cauterize and reduce bleeding in cutaneous surgeries [27,28], intracavitary surgeries (under laparoscopic/thoracoscopic guidance) [29,30,31,32], or conventional laparotomy [33]. The maneuverability of this technique is acceptable, but a unique issue could be the management of the trigger. A complication is the risk of thermal damage to surrounding tissues. In order to avoid this, it is crucial to protect the intestine with a silicone sheet (or similar) and the abdominal wall and liver with the surgeon’s hand. Since these devices can be sterilized, they can be recycled or reused in several surgeries, as in our study, which also benefits the final veterinary costs for the owner [34].

The average surgical time in this study, without including field surgical preparation and anesthesia recovery, was 75–90 min. This is shorter than the laparoscopic technique (99 min) [13], but longer than a conventional omphalectomy. This time can be shortened if cystoplasty is not performed (nevertheless, there were three foals with a patent urachus in the study). However, the authors would recommend performing a cystoplasty in all cases, even in those without a patent urachus, to avoid any future complications related to remnants [35]. Another option to reduce the surgical time is to transect all the vascular structures directly with the LigaSure™, without previous transfixion ligatures. Although the LigaSure™ can be used to safely perform this task, our surgeon preferred to apply the LigaSure™ between double ligations to avoid any risk of contamination of the abdominal cavity, mainly in cases with severe infection or abscessation. Since all surgeries were performed with students in the operation room, the surgeon and the anesthetist spent some extra time for explanations. This could also have increased the time of surgery compared to the conventional technique.

No short- or long-term complications properly related to the modified technique were observed [10,11]; however, a low number of foals were included for further conclusions. The short-term complications in foal number 3 were not related to the surgical procedure, and the foal was euthanized due to septic peritonitis secondary to jejunal rupture by a foreign body, probably from the shaving bed. The foal’s evolution was favorable, and no disturbances of the incision such as infection or dehiscence have been evident to this point (5 days after surgery). Other short-term non-technique-related complications were soft feces and arthritis (septic or immune-mediated). Foal number 2 had arthritis before surgery, probably immune-mediated due to the rapid onset of clinical signs, as well as the laboratory characteristics of the synovial fluid and the lack of bacterial growth in the culture. Thus, it can be assumed that this complication was not directly related to the surgical technique. Foal number 5 underwent a conventional omphalectomy and suffered eventration two days after discharge, but no complications related to the surgical technique had been observed up to then and abnormal ultrasonographic findings had not been observed. The herniation and later contamination of the abdomen due to the reintroduction of eventrated structures could have contributed to the infection of the umbilical vein remnant and liver abscessation [36], although an incomplete vein resection in the initial surgery or a deeper infection in the intrahepatic course of the vein cannot be discarded. This complication could have been avoided if the modified omphalectomy technique had been performed at first instead. Peritoneal response signs were not observed during the necropsy of this foal.

One point to consider in this study was the low number of animals included. However, by incorporating one animal for each of the most common umbilical disorders, the range of possibilities where this modified technique can be used in neonatal foals was covered. It is important to highlight that a greater number of foals were subjected to omphalectomy in our hospital during the study period. However, since there are several teams of surgeons at our facility, each with different preferences, the modified technique is not used in every foal operated on for umbilical disorders in our hospital. In addition, foals that underwent omphalectomy without umbilical abnormalities were not included in the study. Another limitation of this study was that incision lengths were not measured.

Since this is a retrospective study of foals operated on using the modified technique for one year from 1 January 2024, the follow-up period at the time of writing this manuscript is up to 11 months (age of the oldest foal included in the study). Therefore, complications appearing later than this period are unknown at this moment. Although the presence of obstructive intestinal adhesions causing secondary colic cannot be discarded at any time, a period between 5 and 11 months should be sufficient to discard this type of complication, since colic is most commonly observed 2–6 months after laparotomy in foals [10,24,37]. In a similar way, this follow-up period should be enough to notice other common long-term complications, such as hernias [38,39]. Noteworthily, the use of the LigaSure™ device was able to reduce the formation of adhesions between the umbilical vein remnant and surrounding structures. On the other hand, adhesions between the abdominal wall and the intestines should not vary between techniques, since the surgical approach and pattern closure of the abdominal wall are similar between the conventional and modified techniques, but may vary due to incision extension difference between the techniques.

## 5. Conclusions

This case series describes a novel modified two-step omphalectomy technique using the LigaSure™ device in each step for neonate foals with umbilical disorders. Moreover, this is also the first study illustrating this modified technique and showing the umbilical vein abscess disorder in a neonate donkey foal. This modified technique allows removal of the whole extrahepatic portion of the umbilical vein through a shorter midline incision, and could decrease the incidence of future incisional or remnant-related complications. A larger multicentric study including a higher number of foals and a comparative study evaluating omphalectomy techniques are warranted.

## Figures and Tables

**Table 1:** Signalment, clinical signs, and ultrasonographic findings on admission of 5 neonate foals (4 horses and 1 donkey) with umbilical disorders requiring omphalectomy.

Case	Species	Age	Breed	Sex	Anamnesis	Clinical Signs	Ultrasound Findings
1	Donkey	6 d	Andalusian	Female	Total FTPI due to bounding disturbances, urination from the navel. Abnormal laboratory results. On date and normal delivery.	Alert and responsive, good suction reflex, wet and painful stump, no joint disturbances.	Increased and hyperechogenic umbilical vein, open urachus. Pneumonic foci.
2	Horse	12 h	Andalusian	Female	Weakness, abnormal laboratory results.	Weak sucking reflex, poor response to external stimuli, severe fever	Heterogeneous internal umbilical remnant.
3	Horse	12 h	Andalusian	Male	Weakness, no colostrum ingested, premature and assisted labor.	Weakness, weak sucking reflex, hemorrhagic stump, right hindlimb flexural deformity, tacky mucous membranes.	Increased size of both umbilical arteries. Slightly dilated, but motile, small intestine. Meconium impaction.
4	Horse	12 h	Andalusian	Female	Premature, weakness, severe dyspnea. History of perinatal mortalities on the farm.	Severe fever, dyspnea and tachypnea, weak sucking reflex.	Hypoechogenic round images into small colon, abundant comet tails in both hemithorax.
5A	Horse	16 h	Hanoverian	Male	Colic pain and hemorrhagic stump. No meconium expulsion. Delivery on date and unassisted.	Mild colic signs; abdominal distention; thick, painful and hemorrhagic umbilicus; urination from the navel.	Increased size of the umbilical left artery, open urachus, edematous internal umbilical remnant. Meconium impaction.
5B		7 d			Eventration after heavy exercise in the paddock.	Eventration of the omentum.	Midline abdominal wall disruption (approx. 3 cm).

d, days; FTPI, failure of transfer of passive immunity; h, hours. A and B indicate first and second admission to the hospital of foal number 5.

**Table 2:** Diagnosis, short- and long-term post-operative complications, and outcomes of 5 neonate foals (4 horses and 1 donkey) with umbilical disorders undergoing a two-step (using LigaSure^TM^) or conventional omphalectomy.

Case	Diagnosis	Surgical Technique	Short-Term Complications	Time to Discharge (Days)	Long-Term Complications	Outcome
1	Omphalophlebitis with umbilical vein abscess, patent urachus	Modified two-step omphalectomy	None	3	None	Alive, 11 months old
2	Omphalitis, sepsis, total FTPI	Modified two-step omphalectomy	Soft feces (48 h), arthritis and incision seroma	4	Right hock distension	Alive, 5 months old
3	Omphaloarteritis, prematurity, total FTPI, NMS, and meconium impaction	Modified two-step omphalectomy	Enteritis, small-intestine rupture due to a foreign body, and septic peritonitis	*	*	Euthanasia at 11 days post-admission
4	Omphalophlebitis, patent urachus, sepsis, prematurity, interstitial pneumonia, meconium impaction	Modified two-step omphalectomy	-	12	None	Alive, 8 months old
5A	Omphaloarteritis, patent urachus, meconium impaction	Conventional omphalectomy	Soft feces, incisional eventration	5	Incisional eventration	-
5B	Incisional eventration, liver abscess	Modified two-step omphalectomy	Septic polyarthritis, incision infection, diarrhea	*	*	Euthanasia at 27 days post-admission

FTPI, failure of transfer of passive immunity; NMS, neonatal maladjustment syndrome. A and B indicate first and second admission to the hospital of foal number 5. * Foal was not discharged from the hospital.

**Table 3:** Laboratory findings and sepsis score on admission of 5 foals (4 horses and 1 donkey) requiring surgery due to umbilical disorders.

Case	RBCs (×10^6^/μL)	WBCs (×10^3^/μL)	Neutrophils (×10^3^/μL)	Lymphocytes (×10^3^/μL)	Monocytes (×10^3^/μL)	Glucose (mg/dL)	Creatinine (mg/dL)	Lactate (mmol/L)	IgG (mg/dL)	Fibrinogen (mg/dL)	Sepsis Score
1	6.2	8.1	5.8	1.39	0.9	124	1.1	1.8	>800	800	2
2	10.9	3.2	1.1	1.2	0.9	118	6.5	>12	<400	400	13
3	11.0	9.2	7.8	1.3	0.09	90	1.9	6.0	<400	200	11
4	11.5	6.4	5.4	0.7	0.2	120	3.1	>12	>800	600	14
5A	10.9	3.9	2.5	1.3	0.1	173	1.8	6.3	>800	600	8
5B	9.1	16.5	14.4	1.4	0.6	177	1.9	1.1	>800	400	-
Reference range	6.4–10.4	5–11	2.5–6.9	1.5–5.1	0.2–0.6	80–120	0.9–1.7	< 2	>800	<400	<11

Reference ranges correspond to those previously reported in foals, adapted to our animal population [20,21,22]. A and B describe first and second admission to the hospital of foal number 5. RBCs, red blood cells; WBCs, white blood cells.

## Data Availability

The original contributions presented in this study are included in the article/Supplementary Materials. Further inquiries can be directed to the corresponding author.

## Supplementary Materials

The following supporting information can be downloaded at https://www.mdpi.com/article/10.3390/ani15070981/s1. Table S1: Dose and brand marketed products of drugs administered in this study; Video S1: Technique for the LigaSure™ device and surgeon’s hand placement for a complete umbilical vein removal from Case 3.
