# Supplementary material for: Description of a Modified Two-Step Omphalectomy Technique Using the LigaSure™ Device to Remove the Whole Extrahepatic Umbilical Vein: A Case Series Study in Equine and Donkey Foals
Source: Animals (Basel). 2025 Mar 28;15(7):981. doi: 10.3390/ani15070981 (PMC11987979; doi:10.3390/ani15070981)
Supplement: Supplementary file 1 [file animals-15-00981-s001.zip › animals-3496207-supplementary.pdf]

Table S1. Dose and brand marketed products of drugs administered in this study.

Drug	Dose	Route	Interval (hour)	Brand name and manufacturer
Amikacin	25 mg/kg	IV	24	Amikacina, Normon, Madrid, Spain
Ampicillin	20 mg/kg	IV	6	Gobemicina, Normon, Madrid, Spain
Cefquinome	4 mg/kg	IM	12	Cobactan, MSD, Lucerne, Switzerland
Dexamethasone	0.1 mg/kg	IV	24	Cortexona, Syva SA, Leon, Spain
Dexmedetomidine	1 mg/kg	IV	once	Dexdomitor, Orion Pharma, Madrid, Spain
Dobutamine	2 µg/kg/min	IV	CRI	Dobutamine, Hospira, IL, USA
Fentanyl	3 µg/kg	IV	once	Fentanest, Kern Pharma, Barcelona, Spain
Florfenicol	20 mg/kg	IM	24	Nufor, MSD, Lucerne, Switzerland
Flunixin meglumine	0.5-1.1 mg/kg	IV	12	Flunixin injection, Norbrook, Newry, UK
Furosemide	0.1 mg/kg/h	CRI	Upon requirements	Seguril, Sanofi, Paris, France
Ketamine	0.5-1 mg/kg	IV	Upon requirements	Ketamidor, Richter Pharma, Welsm, Austria
Lactase	5000-6000 IU/50 Kg foal	PO	6	Lactofree, Bluecube Healthcare, Cordoba, Spain
Lidocaine	1.3 mg/kg/min	CRI	Upon requirements	Compounded
Magnesium sulphate	20 mg/kg	IV	8	Compounded
Meloxicam	0.6 mg/kg	IV	12	Meloxidyl, Ceva, Marsella, France
Methadone	0.2 mg/kg	IV	once	Metasedin, Esteve Pharmaceuticals SA, Barcelona, Spain
Metronidazole	15 mg/kg	PO	8	Metrobactin, Dechra, Northwich, UK
Midazolam	0.1 mg/kg	IV	once	Midazolam, BBraun, Barcelona, Spain
Misoprostol	4 µg/kg	PO	8	Cytotec, Pfizer, Dublin, Ireland
Propofol	1-3 mg/kg	IV	Upon requirements	Propofol Lipuro, BBraun VetCare, Barcelona, Spain
Sulfadoxine/Trimethoprim	23 mg/kg	IV	12	Borgal, Virbac, Carros, France
Thiamine	5 mg/kg	IV	24	Benerva, Bayer, Leverkusen, Germany
Vitamin E and Selenium	50-75 mg/ 0.25-0.75 mg, 50 Kg foal	IM	72	Selevit complex, Syva SA, Leon, Spain

CRI, constant rate infusion; IM, intramuscular; IV, intravenous; PO, orally.
